# Noncontrast CT-based habitat analysis for differentiating hemorrhagic transformation from contrast extravasation after endovascular therapy of acute ischemic stroke

**DOI:** 10.1186/s41747-026-00798-9

**Published:** 2026-09-10

**Authors:** Yi Huang, Guirong Tan, Weihao Fan, Lijuan Yang, Taotao Zhang, Youyong Zhang, Zongqiang Mai, Jianqing Peng, Huiling Fan, Xiang Liu

**Affiliations:** 1 Department of Interventional Center, Biomedical Innovation Center, The Sixth Affiliated Hospital, Sun Yat-Sen University, Guangzhou, China; 2 Advanced Neuroimaging Laboratory, Yuebei People’s Hospital Affiliated to Shantou University Medical College, Shaoguan, China; 3 Department of Radiology, Yuebei People’s Hospital Affiliated to Shantou University Medical College, Shaoguan, China; 4 Department of Neurology, Yuebei People’s Hospital Affiliated to Shantou University Medical College, Shaoguan, China; 5 Interventional Diagnosis and Treatment Center, Yuebei People’s Hospital Affiliated to Shantou University Medical College, Shaoguan, China; 6 Hepatobiliary and Pancreatic Tumor Treatment Center, Yuebei People’s Hospital Affiliated to Shantou University Medical College, Shaoguan, China; 7 Designated Medical Center, Yuebei People’s Hospital Affiliated to Shantou University Medical College, Shaoguan, China

**Keywords:** Ischemic stroke, Endovascular procedures, Postoperative hemorrhage, Radiomics, Tomography (x-ray computed)

## Abstract

**Objectives:**

Hemorrhagic transformation (HT) is a serious and common complication after endovascular therapy (EVT) in patients with acute ischemic stroke (AIS). This study aimed to evaluate the diagnostic value of habitat analysis in differentiating HT from contrast extravasation (CEx) within hyperdense areas (HDAs) after EVT, and to assess whether the preoperative imaging features provide additional diagnostic value.

**Materials and methods:**

This retrospective study included 187 AIS patients with HDAs. A stratified three-fold cross-validation was used to split the dataset into training and validation cohorts, and preoperative hyperdense artery sign (HAS) status and clinical characteristics were collected. Habitat analysis was performed using the k-means clustering algorithm applied to postoperative noncontrast CT. The radiomics model, habitat model, and corresponding combined models integrating HAS and clinical characteristics were developed. Diagnostic performance was evaluated by the area under the receiver operating characteristic curve (AUC).

**Results:**

The optimal k-value was determined to be 5. In differentiating HT from CEx of HDA, the habitat model achieved the highest AUC values of 0.89 and 0.82 in the training and validation cohorts, respectively. Habitat analysis showed improved diagnostic performance compared with the radiomics analysis (AUCs were 0.81 and 0.75 in the training and validation cohorts). The combined model integrating preoperative imaging features, clinical characteristics, and habitat features did not improve performance.

**Conclusion:**

Habitat analysis outperformed radiomics analysis in differentiating HT from CEx of HDAs after EVT on NCCT. Five‑cluster habitat analysis revealed intralesional heterogeneity within HDAs in AIS patients, supporting its potential as a novel tool for clinical decision‑making.

**Key Points:**

- ***Question*** Differentiation of HT from CEx in post-EVT HDA on non-contrast CT remains a clinical challenge in AIS.
- ***Findings*** Habitat model achieved AUCs up to 0.89 in the training cohort and 0.82 in the validation cohort, outperforming radiomics analysis for differentiating post-EVT HDA and revealed HDA heterogeneity.
- ***Relevance Statement*** Habitat analysis revealed intralesional heterogeneity in the HDA and outperformed radiomics analysis in differential diagnosis on noncontrast CT, which is widely available in most stroke centers. Our findings suggest its potential as a novel clinical decision-making tool.

**Graphical Abstract:**

**Figure:**
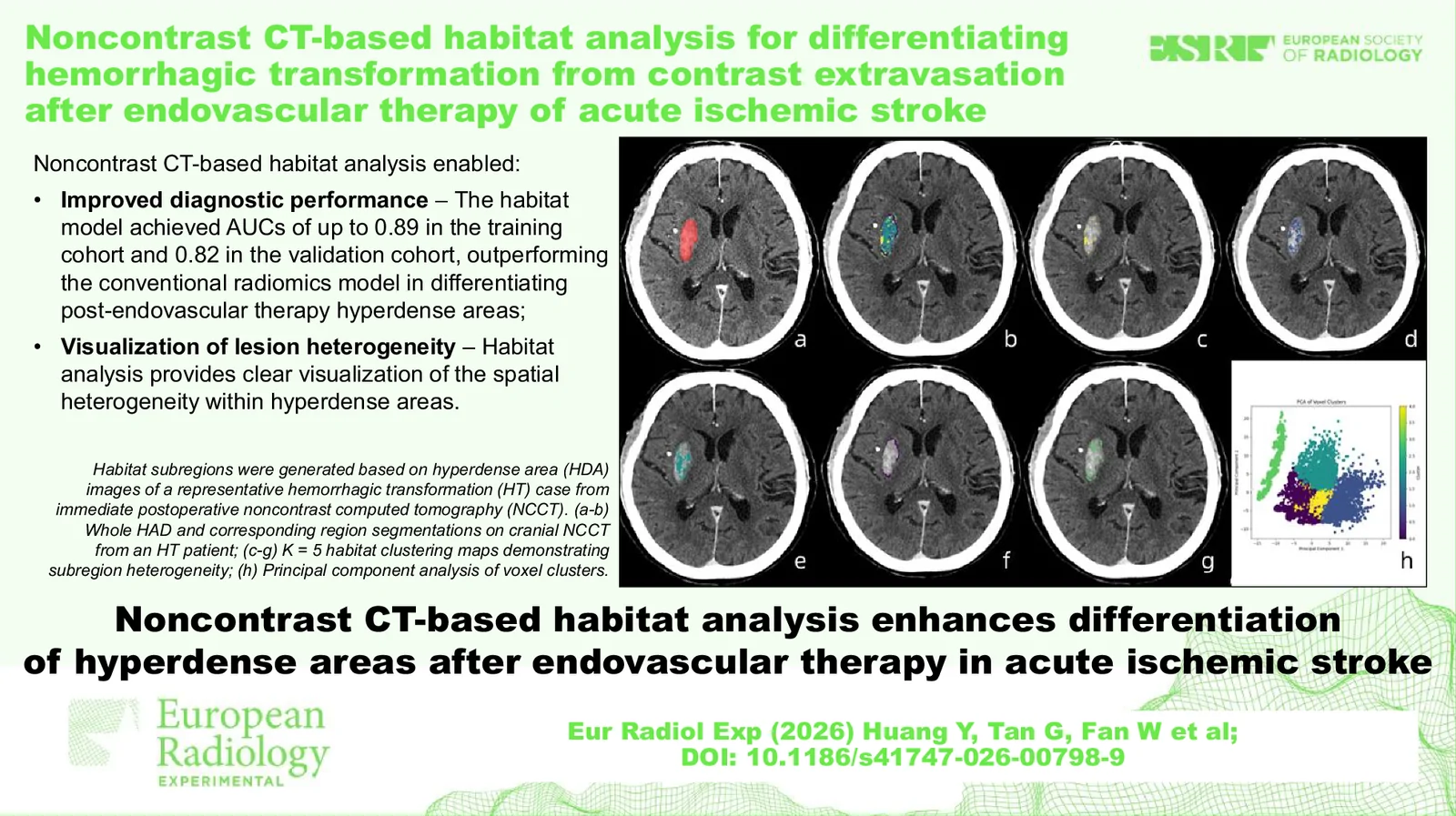

## Background

Acute ischemic stroke (AIS) is a leading global cause of death and is characterized by high incidence, fatality, and disability rates [1]. Endovascular therapy (EVT) with mechanical thrombectomy is the preferred treatment for AIS patients secondary to large vessel occlusions. The hyperdense areas (HDAs) within the cerebral parenchyma on post-procedural noncontrast computed tomography (NCCT) following mechanical thrombectomy are commonly discovered radiological abnormalities, with an incidence of 31.2–87.5% [2]. HDAs may result from contrast extravasation (CEx) or hemorrhagic transformation (HT).

HT is the most serious complication after EVT, leading to a marked deterioration with a mortality of up to 83% [3]. Accurate differentiation between HT and CEx leads to fundamentally different and mutually exclusive treatment strategies. Therefore, early and accurate identification of HDA compositions is essential for guiding post-EVT decisions.

Differentiating between HT and CEx of HDAs on NCCT alone is difficult. In recent years, dual-energy computed tomography (CT) has been reported to distinguish HT from CEx successfully. However, its application is limited by the expensive equipment and low availability. NCCT remains a first-line imaging technique for determining treatment options for AIS [4, 5].

Radiomics extracts high-dimensional quantitative features from medical images [6], and multiple studies have demonstrated its feasibility for guiding diagnosis and treatment in AIS [7–10]. Existing radiomics studies on HDA differentiation remain limited, and the reported diagnostic performance has been variable: Zhai et al [11] applied 2D measurement methods to segment HDA, while some studies suggest that 3D analysis provides richer information. Another study extracted only first-order features from HDA, potentially overlooking risk factors such as second-order features [12]. Chen et al [13] built a radiomics-only model without clinical features. Ma et al [14] achieved the best diagnostic performance; however, their study had a small sample size and risk of overfitting. Hu et al’s study included both parenchymal and subarachnoid hemorrhage as outcome measures and had a high HT prevalence [15]. Moreover, these studies on this topic have mainly relied on radiomic features extracted from the whole lesion. However, HDAs may exhibit substantial heterogeneity within these lesions, which may not be adequately characterized by this strategy [16].

Habitat analysis, which partitions voxels with similar imaging characteristics into distinct subregions, has been shown to better quantify heterogeneity in brain tumors [17]. Compared with whole-lesion radiomics features, habitat analysis may better capture spatial complexity and tissue microenvironmental differences [18]. We believe that intralesional heterogeneity could provide discriminative information, and no prior study has applied habitat analysis to distinguish HT from CEx within HDAs. Therefore, we hypothesized that incorporating habitat features could improve diagnostic performance for this clinically challenging task.

Prior studies of HDAs have focused on NCCT acquired immediately after EVT, relying on single‑time‑point postoperative data without incorporating preoperative imaging information [11–14]. This may omit potentially valuable diagnostic information for differentiating HT from CEx. Currently, no study has combined preoperative and postoperative imaging features for HDA differentiation. In this study, we therefore attempted to integrate preoperative imaging features with postoperative radiomic and habitat features. Among those preoperative features, the hyperdense artery sign (HAS), an important preoperative imaging finding, has been reported to correlate with thrombus histopathological features, stroke etiology, and clinical outcomes [19–22]. A recent radiomics study further showed that HAS may predict post‑EVT prognosis and the risk of HT [23]. However, whether HAS provides additional value in identifying HDA composition remains unknown.

This study aims to evaluate the diagnostic value of radiomics analysis and habitat analysis in differentiating HT from CEx of HDAs after EVT, and investigate whether the combined model incorporating preoperative imaging features can improve the diagnostic performance.

## Materials and methods

### Patient selection

This retrospective study was approved by the ethics committees of the Yuebei People’s Hospital (approval no. YBSKY-2024-200-001), and the requirement for informed consent was waived owing to its retrospective design and the usage of anonymized data.

**Fig. 1:**
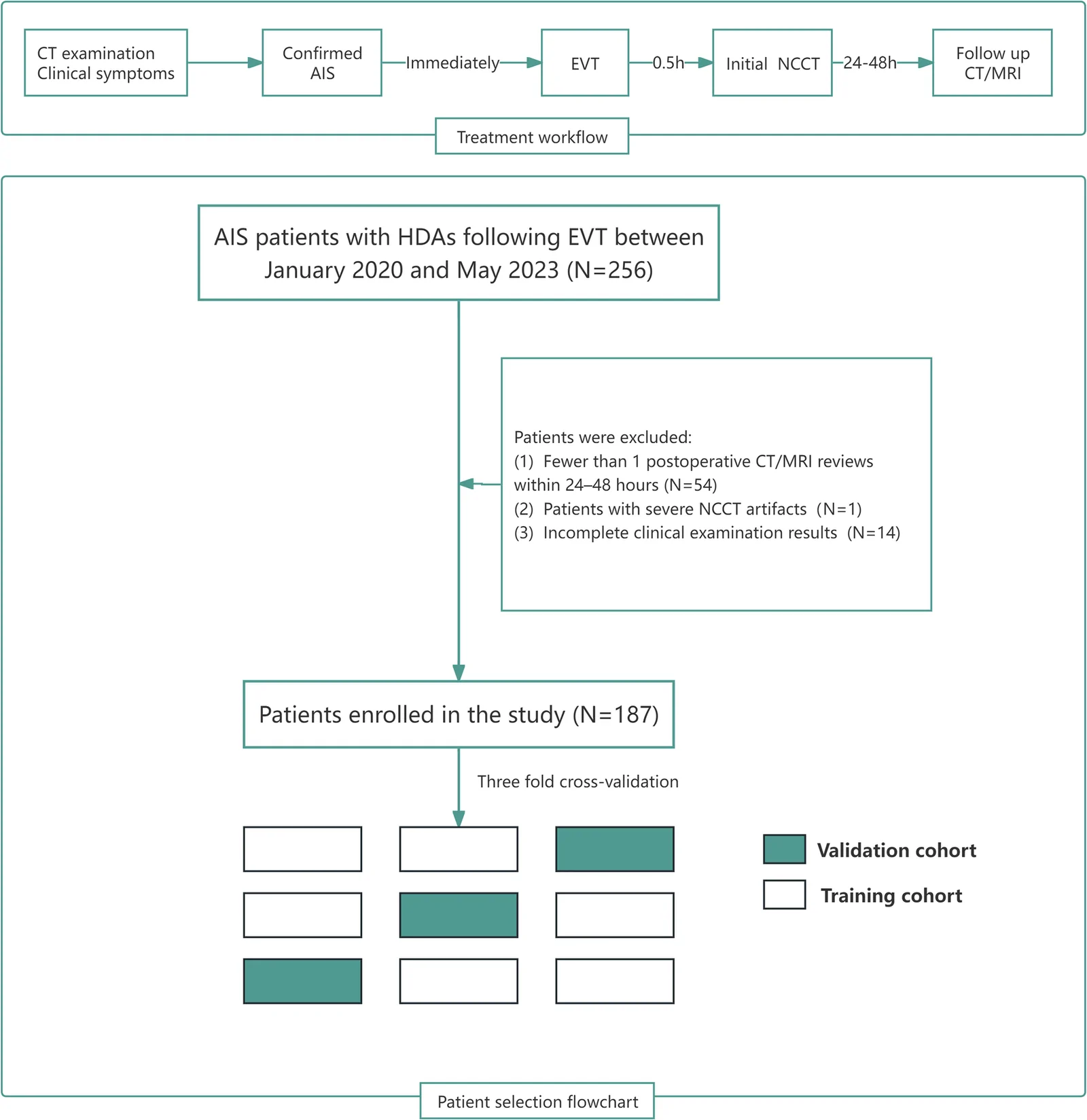
The treatment workflow and patient enrollment flow chart

We retrospectively analyzed the data of patients with AIS who received EVT from January 2020 to May 2023. Patients were included in the study based on the following enrollment criteria: (1) age ≥ 18 years; (2) AIS with large vessel occlusion confirmed by clinical symptoms and imaging; (3) pre-EVT cranial CT to exclude hemorrhage, infection, and tumor; (4) available pre-EVT cranial CT imaging data; (5) receipt of any type of EVT, including mechanical thrombectomy, intra-arterial thrombolysis, balloon angioplasty, stenting, or other mechanical fragmentation; and (6) HDAs detectable on initial cranial NCCT images obtained within 0.5 h after EVT. All enrolled patients received treatment according to current guidelines for AIS, and the dataset was divided into training and validation cohorts using stratified three-fold cross-validation (Fig. 1).

**Fig. 2:**
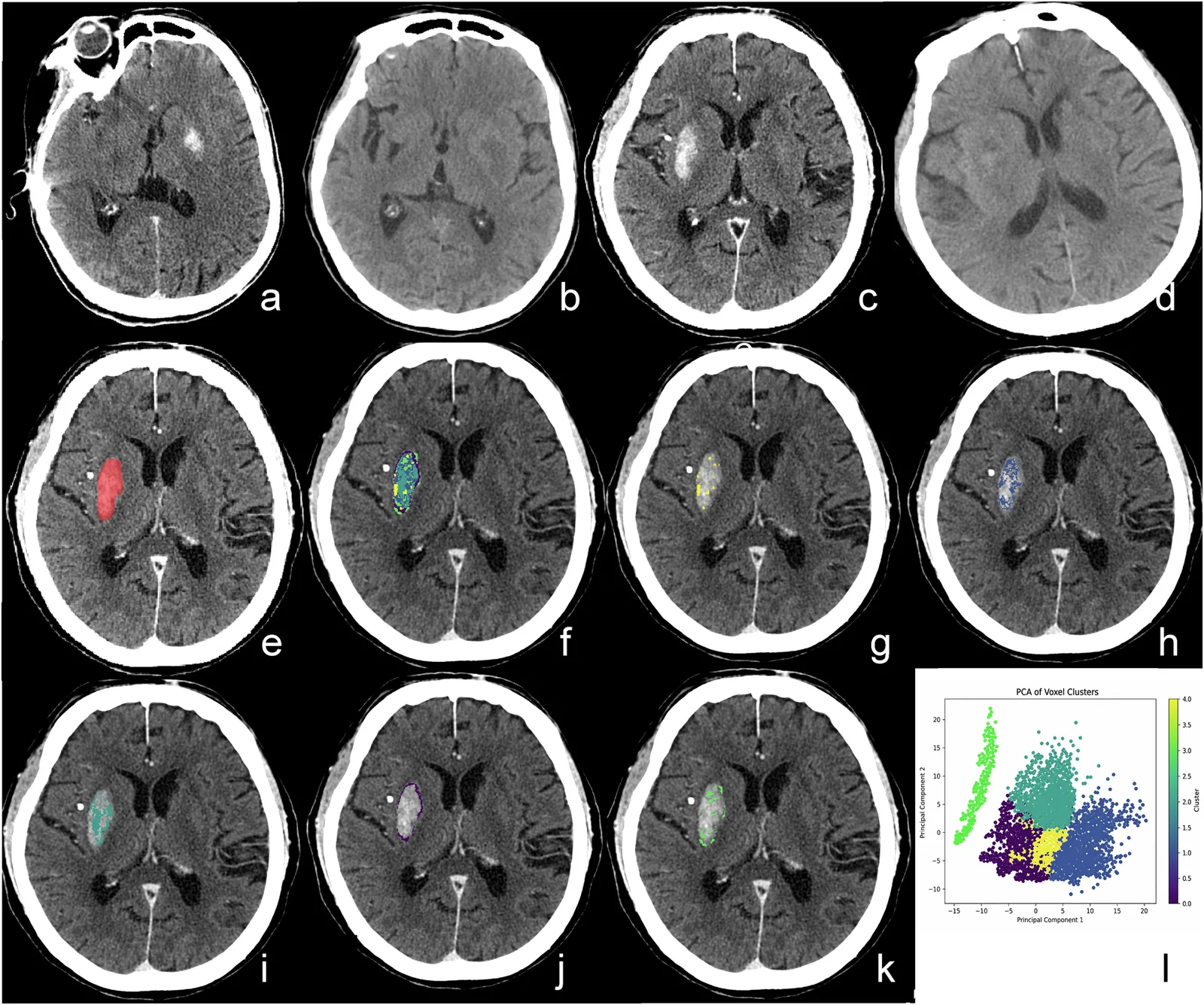
Illustrative cases of CEx, HT, and k = 5 habitat images. First row: Immediate postoperative (**a**) and follow-up (**b**) NCCT in a CEx patient, showing resolution of hyperdensity; immediate postoperative (**c**) and follow-up (**d**) NCCT in an HT patient, showing persistent hyperdensity. Second and third rows: (**e**, **f**) whole HDA and corresponding region segmentations on cranial NCCT from an HT patient; (**g**–**k**) k = 5 habitat clustering maps demonstrating subregion heterogeneity; and (**l**) principal component analysis of voxel clusters. Habitat subregions are color-coded to visualize intralesional heterogeneity: red denotes the entire HDA, while purple, dark blue, green, indigo, and yellow represent the five HDA subregions

HDA was defined as newly developed, nonsolid, visually identifiable parenchymal HDAs measuring ≥ 0.1 cm² without mass effect, and with a density increase of at least 5 HU relative to the contralateral uninvolved brain parenchyma [24]. CEx (non-HT) was defined as the HDA that diminished or resolved completely within 24–48 h [11, 13] (Fig. 2a, b). Conversely, persistence or enlargement of HDA on follow-up imaging indicated HT (Fig. 2c, d).

Early neurologic improvement (ENI) was defined as a reduction of ≥ 8 points in the National Institutes of Health Stroke Scale (NIHSS) score from baseline or an NIHSS score of 0 to 1 within 24 h after thrombectomy [25].

### Clinical and imaging data collection

We collected baseline and preprocedural variables, including demographic data; medical history (*e.g*., hypertension, cardiac disease, diabetes, atrial fibrillation); preoperative intravenous thrombolysis; occlusion site; baseline Alberta Stroke Program Early CT Score (ASPECTS); NIHSS scores (baseline, 2 h postoperatively); systolic/diastolic blood pressure; CT sign: HAS; and Trial of ORG 10172 in Acute Stroke Treatment–TOAST classification. We also collected hematological results, such as baseline blood glucose and hematological indices (hemoglobin, neutrophils, lymphocytes, platelets). All patients underwent EVT under general anesthesia, with femoral or radial artery access selected as the puncture site. Operators recorded the door-to-needle time, puncture-to-reperfusion time, door-to-reperfusion time, EVT duration, number of retrieval attempts, and whether stents were implanted or intra-catheter tirofiban was injected intraoperatively.

NCCT imaging was acquired on United Imaging or GE Healthcare scanners at three time-points: a preoperative scan within 6 h before EVT, an immediate postoperative scan within 0.5 h after EVT, and follow-up CT at 24–48 h. The detailed scanning parameters were presented in Supplementary Table S1. CT images were viewed using fixed head window settings (window level: 40 HU; window width: 80 HU) to minimize interobserver variability during manual lesion delineation.

### HDA segmentation, habitat generation, and feature extraction

Image intensity inhomogeneity was corrected using the N4 bias field correction algorithm, implemented in Simple ITK (Insight Software Consortium, Clifton Park, NY, USA) through Python (version 3.8.0) [26]. All lesions were identified based on HDAs on initial postoperative NCCT images. Regions of interest were manually segmented along lesion contours using ITK-SNAP software (version 4.0.0) by two neuroradiologists (with over 7 and 4 years of experience), who were blinded to patient and clinical information. The radiomics features extracted from the independently delineated ROIs were compared using the intraclass correlation coefficient (ICC), and features with ICC < 0.85 were considered unstable and excluded from further analysis. The HAS was independently assessed on preoperative NCCT images by the same two neuroradiologists, who were also blinded to clinical and outcome data. Each reader recorded the presence or absence of HAS for each case, and inter-reader agreement was quantified using the kappa coefficient.

**Fig. 3:**
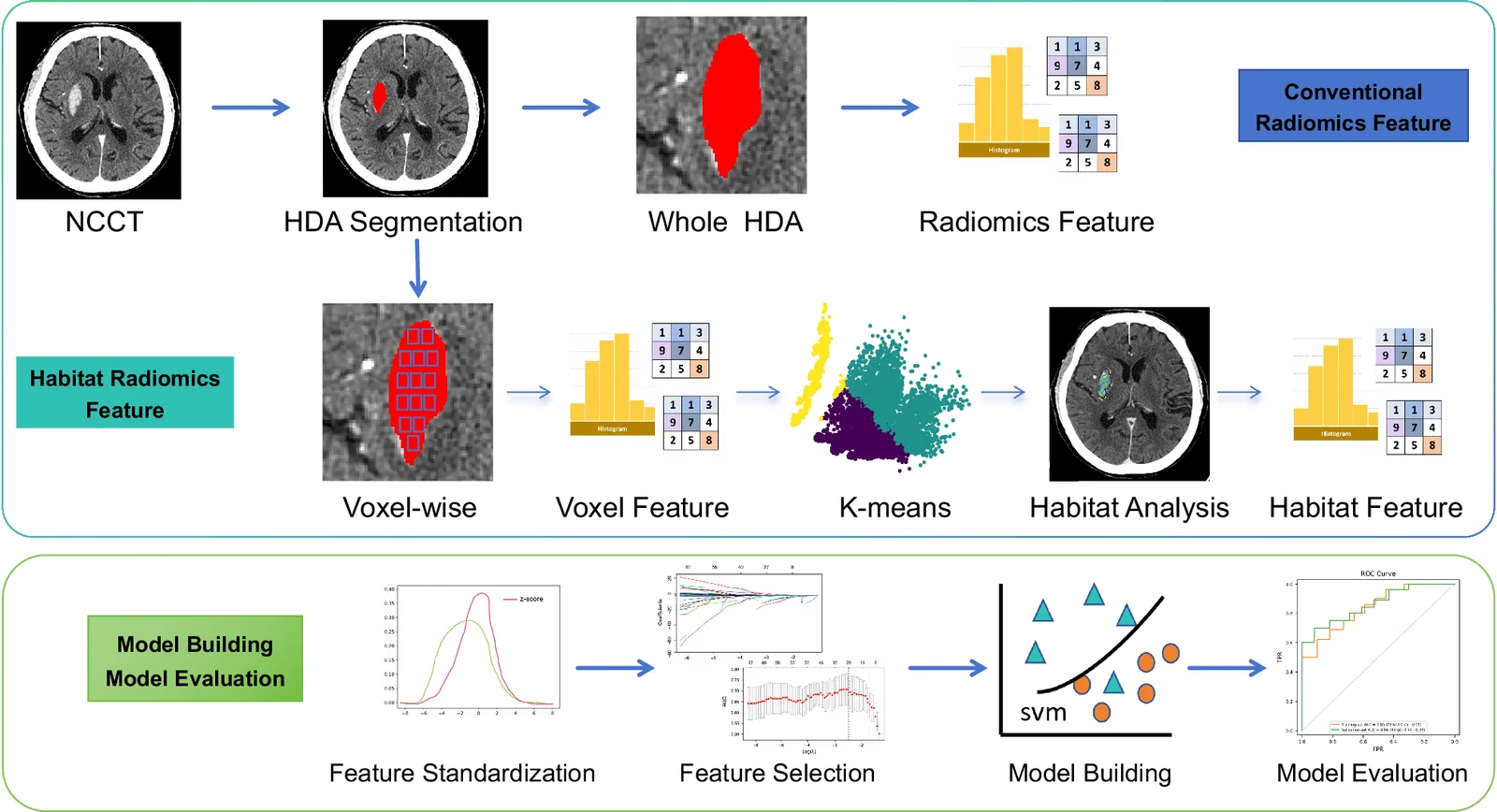
The overall workflow of radiomics and habitat models

Habitat regions were generated via k-means clustering using voxel signal intensities (Fig. 3). Individual voxels were clustered based on squared Euclidean distance between intensity values, with all voxels assigned to distinct spatial habitats in the original image space. The selection of the cluster range of *k* = 2 to 5 was based on prior studies. Figure 2e–k shows the habitat segmentations generated with *k* = 5 for a representative patient with HT. Principal component analysis was applied to reduce the high-dimensional data to a two-dimensional space for visualization. In the resulting principal component analysis plot (Fig. 2l), different colors represent different clusters.

Feature extraction settings were passed in using a configuration YAML file as described in the PyRadiomics documentation (Supplemental Document 1). A total of 1,231 radiomic features were extracted by using PyRadiomics (https://github.com/Radiomics/pyradiomics), including first-order statistics, shape-based features, gray level co-occurrence matrix—GLCM, gray level size zone matrix—GLSZM, gray level run length matrix—GLRLM, gray level dependence matrix—GLDM, and wavelet-based features. For radiomics analysis, features were extracted from the entire HDA volume; whereas for habitat analysis, features were extracted from each subregion.

### Radiomics and habitat model construction

Before selecting radiomics and habitat features, *z*-score normalization was applied to all features. Within each training fold, the Mann–Whitney *U*-test was used to determine the significantly different features. The selected features were subsequently entered into the least absolute shrinkage and selection operator–LASSO regression with ten-fold cross-validation, and features with non-zero coefficients at the optimal λ value were retained as the most informative features. Rad-score and HDA heterogeneity were generated by linearly weighting the selected features according to their corresponding least absolute shrinkage and selection operator coefficients.

Based on the Rad-score or HDA heterogeneity, support vector machine (SVM) models with a linear kernel were constructed. To address class imbalance in the training cohort, class weights inversely proportional to class frequencies were incorporated into the SVM model. Hyperparameter optimization was performed using a grid-search strategy over a predefined cost parameter range (*C* = 2^-5^ to 2^5^), combined with tenfold cross-validation within the training data. The optimal value of the cost parameter (C) was selected based on cross-validation performance. Finally, the validation cohort was used to evaluate the performance of the optimized SVM model. The workflow of radiomics/habitat processing and model construction is shown in Fig. 3.

### Clinical and CT sign characteristics analysis and model construction

Univariate and multivariate logistic regression analyses were applied to identify the independent risk factors of HT. Variables with *p* < 0.050 in multivariate analysis were considered independent risk characteristics. In this analysis, independent clinical features were used to construct clinical models, and independent CT sign (HAS) was used to construct CT-sign (HAS) models.

### Construction and validation of a combined model

Based on the different feature sets, a series of models were constructed using logistic regression: (1) clinical-CT sign (HAS); (2) radiomics-clinical; (3) radiomics-CT sign (HAS); and (4) a combination of clinical, CT sign (HAS), and radiomics. This same modeling strategy was then repeated in the habitat analysis: (1) clinical-CT sign (HAS); (2) habitat-clinical; (3) habitat-CT sign (HAS); and (4) a combination of clinical, CT sign (HAS), and habitat. The performance of the different models was evaluated using the area under the receiver operating characteristic curve (AUC) of receiver operating characteristic, accuracy, sensitivity, and specificity.

### Calibration curve analysis and decision

Calibration curves were plotted for the training and validation cohorts to assess the agreement between predicted and actual risks. Decision curve analysis was also conducted on both cohorts to evaluate the clinical utility of the model by measuring its net benefit across different threshold probabilities.

### Statistical analysis

All statistical analyses were performed using R software (version 4.2.2). Standardization (*z*-score normalization) was applied to balance the variance of the CT sign and all clinical characteristics. Quantitative variables consistent with a normal distribution were presented as mean ± standard deviation. Otherwise, the median (interquartile range) was used. Categorical variables as counts and percentages. The differences between subgroups of categorical variables were assessed using χ^2^ or Fisher's exact tests, while the *t*-test or Wilcoxon test was used for comparisons between quantitative variables.

## Results

### Preoperative characteristics between the HT and non-HT cohorts

Overall, 187 patients were enrolled, of whom 60 (32.09%) had HT. The median age was 66.50 years, and 58 (31.02%) were female. For HAS assessment, the inter-reader κ coefficient was 0.881, indicating excellent inter-observer agreement. Within the HT group, 10 cases were classified as hemorrhagic infarction 1 (HI 1), 16 as hemorrhagic infarction 2 (HI 2), 22 as parenchymal hemorrhage type 1, and 12 as parenchymal hemorrhage type 2. Significant differences were observed between the HT and non-HT groups in baseline ASPECTS, ENI, serum magnesium, serum chloride, and HAS (*p* < 0.050) (Table 1).

**Table 1:** Comparison of baseline characteristics between the HT and non-HT cohorts

Characteristics	All patients (*n* = 187)	HT (*n* = 60)	non-HT (*n* = 127)	*p-*value
Age (median [IQR])	66.50 [58.00, 72.75]	67.50 [57.000, 76.00]	66.00 [59.00, 71.00]	0.356
Female, *n* (%)	58 (31.02)	22 (36.67)	36 (28.35)	0.328
Hypertension, *n* (%)	122 (65.24)	38 (63.33)	84 (66.14)	0.832
Atrial fibrillation, *n* (%)	70 (37.43)	27 (45.00)	43 (33.86)	0.191
Cardiac disease, *n* (%)	53 (28.34)	20 (33.33)	33 (25.98)	0.386
Previous stroke/TIA, *n* (%)	22 (11.76)	5 (8.33)	17 (13.39)	0.449
Smoking, *n* (%)	62 (33.16)	16 (26.67)	46 (36.22)	0.840
Drinking, *n* (%)	21 (11.23)	4 (6.67)	17 (13.39)	0.705
Baseline NIHSS (median [IQR])	15.00 [10.00, 21.00]	15.50 [12.00, 21.00]	15.00 [10.00, 21.00]	0.479
Baseline ASPECTS (median [IQR])	8.00 [7.00, 8.00]	8.00 [7.00, 8.00]	8.00 [7.00, 8.00]	0.024^*^
Baseline SBP (median [IQR])	156.00 [136.00, 177.50]	155.50 [134.75, 171.50]	158.00 [136.50, 178.00]	0.586
Baseline DBP (median [IQR])	93.00 [85.00, 101.50]	96.00 [84.00, 102.75]	92.00 [85.00, 101.00]	0.802
Glucose (median [IQR])	6.73 [5.31, 8.90]	7.33 [5.59, 9.19]	6.44 [5.23, 8.72]	0.125
LDL (median [IQR])	2.72 [2.12, 3.26]	2.61 [2.13, 3.16]	2.79 [2.13, 3.31]	0.614
HDL (median [IQR])	1.09 [0.94, 1.25]	1.14 [1.01, 1.25]	1.07 [0.93, 1.24]	0.270
TC (median [IQR])	4.41 [3.82, 5.17]	4.27 [3.81, 5.06]	4.46 [3.83, 5.23]	0.548
TG (median [IQR])	1.06 [0.77, 1.40]	0.99 [0.73, 1.34]	1.12 [0.80, 1.44]	0.285
Serum magnesium (median [IQR])	0.83 [0.76, 0.89]	0.86 [0.79, 0.93]	0.82 [0.75, 0.87]	0.028^*^
Serum chloride (median [IQR])	105.00 [103.00, 107.00]	104.20 [103.00, 106.50]	105.80 [104.00, 108.00]	0.039^*^
Anion gap	13.60 [11.80, 15.50]	13.87 [12.37, 15.76]	13.57 [11.60, 15.50]	0.323
HGB (median [IQR])	129.00 [115.00, 141.75]	132.50 [114.75, 144.25]	127.00 [115.00, 141.00]	0.514
PT (median [IQR])	12.00 [11.20, 12.80]	12.25 [11.60, 13.13]	11.90 [11.20, 12.70]	0.131
TT (median [IQR])	18.15 [17.20, 19.43]	18.50 [17.40, 19.95]	18.00 [17.20, 19.30]	0.168
INR (median [IQR])	1.02 [0.96, 1.10]	1.02 [0.96, 1.10]	1.02 [0.96, 1.10]	0.065
FIB (median [IQR])	2.76 [2.23, 3.45]	2.61 [2.03, 3.12]	2.85 [2.27, 3.56]	0.091
PLT (median [IQR])	219.00 [184.00, 257.00]	210.50 [167.75, 247.00]	220.50 [190.25, 261.00]	0.059
HbA1c (median [IQR])	5.29 [3.90, 7.20]	6.00 [5.70, 6.40]	5.90 [5.60, 6.60]	0.414
PLR (median [IQR])	172.53 [131.91, 226.04]	163.50 [125.43, 202.50]	180.17 [134.97, 236.88]	0.060
NLR (median [IQR])	5.59 [3.92, 8.39]	5.58 [4.24, 8.26]	5.59 [3.78, 8.48]	0.894
TOAST				0.162
LAA, *n* (%)	94 (50.27)	27 (45.00)	67 (52.76)	
CE, *n* (%)	64 (34.22)	24 (40.00)	40 (31.50)	
SUE, *n* (%)	16 (8.56)	5 (8.33)	11 (8.66)	
SOE, *n* (%)	9 (4.81)	1 (1.67)	8 (6.30)	
SAA, *n* (%)	4 (2.14)	3 (5.00)	1 (0.79)	
Occlusion site				0.555
ICA, *n* (%)	51 (27.2%)	17 (28.33)	34 (26.77)	
MCA, *n* (%)	85 (45.6%)	24 (40.00)	61 (48.03)	
Posterior circulation, *n* (%)	30 (16.0%)	9 (15.00)	21 (16.54)	
Tandem lesions, *n* (%)	21 (11.2%)	10 (16.67)	11 (8.66)	
Tirofiban, *n* (%)	75 (40.11)	20 (33.33)	55 (43.31)	0.255
IVT, *n* (%)	43 (22.99)	16 (26.67)	27 (21.26)	0.526
Thrombectomy status				0.114
No, *n* (%)	73 (39.04)	18 (30.00)	55 (43.31)	
Yes, *n* (%)	114 (60.96)	42 (70.00)	72 (56.69)	
HAS				0.007^*^
Yes, *n* (%)	101 (54.01)	41 (68.33)	60 (47.24)	
No, *n* (%)	86 (45.99)	19 (31.67)	67 (52.76)	
DNT (median [IQR])	104.00 [55.00, 219.50]	94.00 [55.00, 151.50]	121.00 [53.00, 237.50]	0.356
DRT (median [IQR])	123.00 [82.50, 197.00]	111.50 [71.75, 195.00]	123.00 [87.00, 202.00]	0.389
PRT (median [IQR])	35.00 [23.00, 60.00]	34.00 [22.00, 62.00]	35.00 [24.00, 59.50]	0.376
Operation duration (median [IQR])	80.00 [50.00, 105.00]	76.00 [49.25, 100.00]	80.00 [50.00, 110.00]	0.523
ENI				< 0.001^*^
Yes, *n* (%)	117 (62.57)	28 (46.67)	89 (70.08)	
Stable, *n* (%)	37 (19.79)	11 (18.33)	26 (20.47)	
No, *n* (%)	33 (17.65)	21 (35.00)	12 (9.45)	
HT	60 (32.09)	60 (100)	-	-
HI 1, *n* (%)	10 (5.35)	10 (16.67)	-	
HI 2, *n* (%)	16 (8.56)	16 (26.67)	-	
PH 1, *n* (%)	22 (11.76)	22 (36.67)	-	
PH 2, *n* (%)	12 (6.42)	12 (20)	-	
CT scanners				0.697
United imaging, *n* (%)	142 (75.94)	44 (73.33)	98 (77.17)	
GE, *n* (%)	45 (24.06)	16 (26.67)	29 (22.83)	
EVT				0.158
1	99 (52.94)	39 (65.00)	60 (47.24)	
2	19 (10.16)	3 (5.00)	16 (12.60)	
3	53 (28.34)	13 (21.67)	40 (31.50)	
4	15 (8.02)	5 (8.33)	10 (7.87)	
5	1 (0.53)	0 (0.00)	1 (0.79)	

*1* = Thrombectomy, *2* = Thrombectomy + balloon dilation, *3* = Thrombectomy + balloon dilation + stent implantation procedure, *4 =* Thrombectomy + stent implantation, 5 = Balloon dilation + stent implantation

* p < 0.05, *ASPECTS* Alberta Stroke Program Early Computed Tomography Score, *CE* Cardioembolism, *DBP* Diastolic blood pressure, *DNT* Door-to-needle time, *DRT* Door-to-reperfusion time, *ENI* Early neurologic improvement, *EVT* Endovascular therapy, *FIB* Fibrinogen, *HAS* Hyperdense artery sign, *HbA1c* Hemoglobin A1c, *Hb* Hemoglobin, *HI 1* Hemorrhagic infarction type 1, *HI 2* Hemorrhagic infarction type 2, *HDL* High-density lipoprotein, *HT* Hemorrhagic transformation, *ICA* Internal carotid artery, *INR* International normalized ratio, *IVT* Intravenous Thrombolysis, *LAA* Large artery atherosclerosis, *LDL* Low-density lipoprotein, *MCA* Middle cerebral artery, *NIHSS* National institute of health stroke scale, *NLR* Neutrophil-to-lymphocyte ratio, *PH 1* Parenchymal hemorrhage type 1, *PH 2* Parenchymal hemorrhage type 2, *PLR* Platelet-to-lymphocyte ratio, *PLT* Platelet, *PRT* Puncture-to- reperfusion time, *PT* Prothrombin time, *SBP* Systolic blood pressure, *SOE* Stroke of other determined etiology, *SUE* Stroke of undetermined etiology, *TC* Total cholesterol, *TG* Triacylglycerol, *TIA* Transient ischemic attack, *TOAST* Trial of Org 10,172 in Acute Stroke Treatment, *TT* Thrombin time

### Logistic regression analysis

To ensure robustness, three separate logistic regression analyses were performed using training cohorts derived from three-fold cross-validation. Here, we present the results of one representative analysis; the results of the other two analyses are provided in Supplementary Table S2. As shown in Table 2, univariate and multivariate logistic regression analyses identified baseline ASPECTS and ENI as independent predictors of HT (both *p* < 0.050). Patients with postoperative ENI (OR = 0.07, 95% CI: 0.01–0.33) and higher baseline ASPECTS (OR = 0.61, 95% CI: 0.38–0.92) exhibited a significantly lower risk of HT.

**Table 2:** Logistic regression analysis for independent predictors in the training cohort

Variables	Univariate analysis		Multivariate analysis	
	OR (95%CI)	*p*-value	OR (95%CI)	*p*-value
Baseline ASPECTS	0.59 (0.39–0.86)	0.007^*^	0.61 (0.38–0.93)	0.027^*^
ENI				
No	-	-	-	-
Stable	0.15 (0.05–0.41)	< 0.001^*^	0.16 (0.04–0.51)	0.002^*^
Yes	0.25 (0.04–0.81)	0.025^*^	0.07 (0.01–0.33)	0.001^*^
TC	0.63 (0.40–0.94)	0.030^*^	0.66 (0.39–1.05)	0.089
Thrombectomy status				
No	-	-	-	-
Yes	2.8 (1.25–6.68)	0.016^*^	2.37 (0.92–6.46)	0.080

* p < 0.05, *ASPECTS* Alberta Stroke Program Early Computed Tomography Score, *CI* Confidence interval, *ENI* Early neurologic improvement, *OR* Odds ratio, *TC* Total cholesterol

### Performance of the models

The ICC for the extracted features was good (ICC > 0.86). As shown in Table 3, for clinical analysis, the mean AUC of the clinical model was 0.72 (95% CI: 0.63–0.82) in the training cohort and 0.53 (95% CI: 0.37–0.70) in the validation cohort. The CT-sign model yielded a mean AUC of 0.61 (95% CI: 0.52–0.70) in the training cohort and 0.61 (95% CI: 0.48–0.73) in the validation cohort. After further integrating clinical characteristics, the clinical-CT sign model showed enhanced diagnostic ability, with a mean AUC of 0.75 (95% CI: 0.67–0.84) in the training cohort and 0.61 (95% CI: 0.45–0.77) in the validation cohort.

**Table 3:** The performance of different models in differentiating HDAs

Model	Cohort	Mean AUC	95% CI	ACC	SN	SP
Radiomics						
Radiomics model	Training	0.81	0.73–0.89	0.71	0.74	0.70
	Validation	0.75	0.62–0.88	0.65	0.68	0.64
Clinical model	Training	0.68	0.58–0.77	0.71	0.51	0.81
	Validation	0.63	0.48–0.78	0.66	0.42	0.77
CT sign (HAS) model	Training	0.61	0.51–0.70	0.58	0.68	0.53
	Validation	0.61	0.48–0.73	0.58	0.68	0.53
Clinical-CT sign (HAS) model	Training	0.73	0.64–0.83	0.68	0.62	0.71
	Validation	0.70	0.55–0.84	0.62	0.55	0.65
Radiomics-Clinical model	Training	0.84	0.77–0.92	0.69	0.85	0.61
	Validation	0.8	0.70–0.91	0.6	0.75	0.54
Radiomics-CT sign (HAS) model	Training	0.81	0.73–0.89	0.69	0.71	0.69
	Validation	0.75	0.63–0.88	0.66	0.67	0.65
Combined model	Training	0.85	0.78–0.92	0.73	0.73	0.73
	Validation	0.80	0.69–0.91	0.73	0.73	0.72
Habitat (*k* = 5)						
Habitat model	Training	0.89	0.83–0.95	0.8	0.78	0.81
	Validation	0.82	0.71–0.93	0.72	0.65	0.75
Clinical model	Training	0.72	0.63–0.82	0.65	0.63	0.67
	Validation	0.53	0.37–0.70	0.58	0.42	0.65
CT sign (HAS) model	Training	0.61	0.52–0.70	0.58	0.68	0.53
	Validation	0.61	0.48–0.73	0.58	0.68	0.53
Clinical-CT sign (HAS) model	Training	0.75	0.67–0.84	0.69	0.58	0.75
	Validation	0.61	0.45–0.77	0.66	0.47	0.75
Habitat-Clinical model	Training	0.91	0.86–0.97	0.79	0.83	0.78
	Validation	0.77	0.65–0.90	0.69	0.63	0.71
Habitat-CT sign (HAS) model	Training	0.89	0.83–0.95	0.79	0.79	0.79
	Validation	0.82	0.70–0.93	0.73	0.72	0.74
Combined model	Training	0.92	0.87–0.96	0.81	0.81	0.81
	Validation	0.78	0.65–0.90	0.71	0.6	0.76

*ACC* Accuracy*, AUC* Area under the curve of the receiver operating characteristic curve*, CI* Confidence interval*, CT* Computed tomography*, HAS* Hyperdense artery sign*, SN* Sensitivity*, SP* Specificity

In radiomics analysis, the radiomics model achieved optimal performance with mean AUC values of 0.81 (95% CI: 0.73–0.89) in the training cohort and 0.75 (95% CI: 0.62–0.88) in the validation cohort. The combined model yielded comparable results, with a mean AUC of 0.85 (95% CI: 0.78–0.92) and 0.80 (95% CI: 0.69–0.91) in the training and validation cohorts, respectively. For habitat analysis, the optimal cluster number was determined to be 5 based on the highest mean AUC value (Supplementary Table S3). The *k* = 5 habitat models demonstrated superior diagnostic performance, achieving a mean AUC of 0.89 (95% CI: 0.83–0.95) in the training cohort and 0.82 (95% CI: 0.71–0.93) in the validation cohort. The addition of clinical variables and HAS provided negligible predictive benefit, as the combined model did not significantly outperform the habitat model alone, reaching a mean AUC of 0.92 (95% CI: 0.87–0.96) and 0.78 (95% CI: 0.65–0.90) (The mean AUCs in Table 3, the detailed AUCs from each fold are stored in Supplemengtary Table S4).

**Fig. 4:**
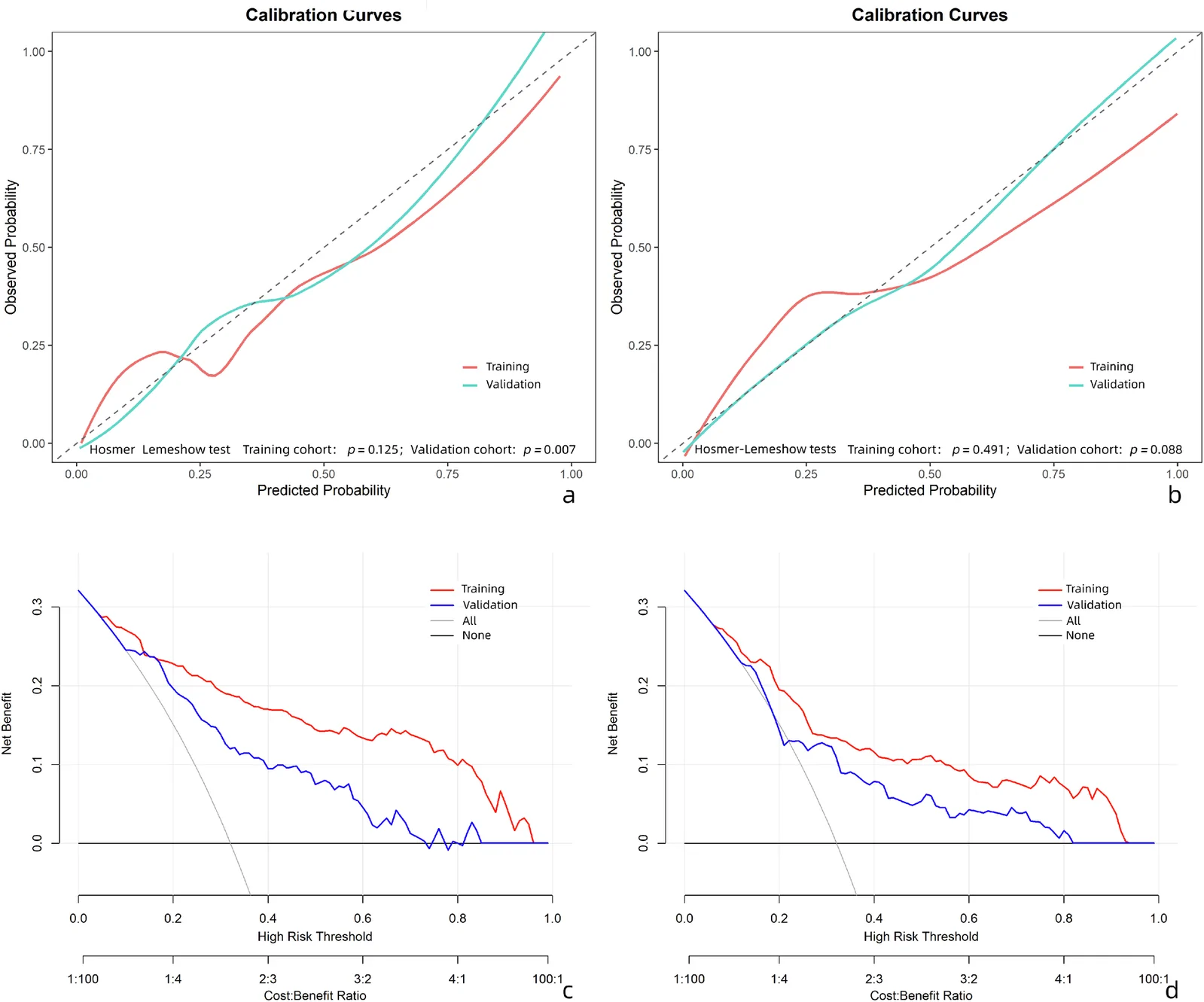
Calibration curves and decision curves of the radiomics analysis model and the habitat analysis model (k = 5). The calibration curves of the radiomics model (**a**) and habitat model (**b**) reflect the consistency between model-predicted probabilities and actual clinical outcomes. The decision curves in  the radiomics model (**c**) and the habitat model (**d**) cohorts clarify the clinical implications of false-positive and false-negative results of these models, which helps assess their net clinical benefit

In both the training cohort and validation cohort, calibration curves and Hosmer-Lemeshow tests (*p* = 0.491, *p* = 0.088; Fig. 4a, b) revealed good agreement between the predicted and observed probabilities of the habitat and radiomics models. The decision curve analysis (Fig. 4c, d) demonstrated that the radiomics and the habitat models provided a higher net benefit than the comparison models. These findings supported the clinical utility of the habitat model.

## Discussion

In this study, 60 patients (32.09%) developed HT. The optimal number of clusters (k) was determined as 5. Our findings suggested that habitat analysis could effectively differentiate HT from CEx of HDA after EVT, achieving the best mean AUC values of 0.89 in the training cohort and 0.82 in the validation cohort, which were higher than the AUC values of the radiomics models (0.81 and 0.75, respectively). The combined model, which integrated clinical characteristics, HAS, and habitat features, achieved mean AUC values of 0.92 (training cohort) and 0.78 (validation cohort), confirming that these additional variables provided no improvement over the habitat model alone.

Our results are comparable to those reported in previous radiomics studies on HDA [11,13], supporting the credibility and robustness of our study. For instance, Zhai et al [11] reported an external validation AUC of 0.798, and Chen et al [13] achieved an AUC of 0.826. While Ma et al obtained higher AUCs (0.955 and 0.869), that study reported a notably high HT prevalence (73.08%) [14]. In contrast, the HT rate in our cohort (32.09%) is consistent with the 30.7% reported in a meta‑analysis by Sun et al [27] and may better reflect real-world prevalence. Whether HT prevalence influences the diagnostic performance of radiomics models remains unclear and warrants further evaluation.

Habitat analysis achieved mean AUC values of 0.89 in the training cohort and 0.82 in the validation cohort, indicating that the habitat-based subregional approach is feasible for discrimination between HT and CEx. To the best of our knowledge, this is the first study to apply habitat analysis to post-EVT HDAs. Compared with the radiomics model, the habitat model demonstrated superior diagnostic performance. This improvement may be attributed to the ability of habitat analysis to capture intra-lesional heterogeneity that whole-lesion radiomics cannot provide. Although the observed AUC gain remains modest, habitat analysis provides a novel research approach and direction.

Beyond diagnostic performance, accurate differentiation of HT from CEx holds direct therapeutic implications. If the model assessment favors HT, this would suggest deferring antithrombotic therapy and arranging prompt confirmatory imaging (*e.g*., dual-energy CT or susceptibility‑weighted MRI) without awaiting the 24‑h follow‑up. In contrast, the assessment favoring CEx would support earlier consideration of antithrombotic therapy, in conjunction with preoperative ASPECTS and postoperative ENI assessment. Furthermore, timely and accurate differentiation of HT from CEx by NCCT‑based habitat analysis enables early implementation of targeted medical interventions, such as appropriate pharmacological management or intensive blood pressure control, which may ultimately help improve patient prognosis. Considering the exploratory nature of this work, the model is not yet intended for direct clinical decision‑making, and its clinical utility awaits prospective validation.

Habitat subregions are commonly generated through clustering algorithms, among which k‑means clustering is the most widely adopted unsupervised technique for partitioning lesions into distinct subregions [28]. However, the optimal cluster number remains inconsistent across studies. In the present study, we tested cluster numbers ranging from 2 to 5 and found that the *k* = 5 model achieved the highest diagnostic performance, consistent with the *k* = 5 setting reported in some oncologic habitat studies [29–31], whereas other values, including 2, 3, and 4, as well as arbitrary selections, have also been described [32–36]. In brain tumor research, *k* = 2 and *k* = 3 are the most frequently adopted [36–38], and the limited number of habitat studies in stroke has predominantly used *k* = 2 for thrombus evaluation [19]. Given this variability, the optimal cluster number likely depends on the specific clinical context and the underlying tissue composition of the target lesion.

The biological interpretation of habitat analysis remains a challenge, as the association between habitat features and underlying tissue biology is not fully understood [28]. In the context of post-EVT HDAs, the biological identity of the habitat subregions remains speculative. As no histopathological confirmation of their tissue characteristics has been reported in humans to date, nor could these subregions be validated by dual-energy CT. In the present study, the k = 5 cluster model demonstrated a finer characterization of intra-lesional heterogeneity as evidenced by its superior diagnostic performance. Accordingly, we hypothesize that the five subregions may correspond to HT, CEx, and their various combinations with normal brain parenchyma, potentially reflecting a spectrum of blood‑brain barrier (BBB) injury rather than discrete pathological entities. Specifically, further damage to the endothelial extracellular matrix in the core infarct region could increase BBB permeability to varying degrees, permitting the extravastion of contrast material and blood components such as red blood cells and thereby contributing to the various combinations described above. This interpretation, however, remains strictly speculative, and the definitive biological identity of these subregions requires future experimental validation, including animal models with histological confirmation.

To the best of our knowledge, this study is the first to combine preoperative imaging features of HAS with postoperative radiomic and habitat features for HDA differentiation, reflecting our research direction that integrates pre‑ and post‑procedural imaging information. However, HAS did not improve the diagnostic performance of the habitat model. This lack of benefit likely reflects a fundamental mismatch: HAS, as a thrombus‑attribute marker, captures initial thrombus composition and ischemic severity rather than the post‑reperfusion tissue state that determines HT *versus* CEx. Other variables, such as recanalization status, BBB integrity, and peri‑procedural blood pressure fluctuations, further decouple the thrombus signal from the ultimate HDA outcome. Moreover, the current HAS assessment relies on subjective qualitative visual evaluation without standardized density thresholds, which may introduce classification errors. In the present study, HAS was used as a pre-operative imaging feature because it indicates large vessel occlusion, and it has been shown to correlate with the histological composition of thromobolus, and is associated with various stroke etiology. While the added predictive value of HAS was limited in this study, the proposed strategy of integrating preoperative and postoperative imaging features represents a novel direction and may inform future research. Future studies should explore quantitative radiomic or habitat features of HAS to strengthen its utility in diagnostic models.

This study has several important limitations. First, the retrospective, single‑center design and relatively small sample size may introduce potential selection bias and restrict the generalizability of the conclusions. Second, the observational endpoint used to differentiate HT from CEx was based on conventional criteria applied in most existing studies; whether this classification affects model performance requires further investigation. Third, despite the use of stratified three‑fold cross‑validation, the high dimensionality of radiomic features relative to the limited sample size still carries the risk of overfitting. Fourth, although good inter‑reader agreement was achieved with manual segmentation, observer dependence cannot be fully eliminated. Fifth, the lack of histopathological validation prevents a definitive biological interpretation of the habitat subregions. Future investigations should employ prospective multicenter designs with larger patient cohorts, external validation, and integration with advanced imaging or histopathological correlation to further validate and establish the clinical utility of habitat analysis.

This study showed that the habitat analysis outperforms radiomics analysis in distinguishing HT from CEx in HDAs on NCCT. It may serve as a novel tool in this field and may thereby facilitate prompt and appropriate clinical decision-making. The five-cluster habitat analysis revealed intralesional heterogeneity within HDAs, providing speculative insights into their underlying characteristics.

## Supplementary information

ELECTRONIC SUPPLEMENTARY MATERIAL **SupplementaryTable S1** Scanning parameters of three CT scanners in this study. **Supplementary Table S2-1** Univariate and multivariate logistics regression analysis of the training cohort in habitat analysis (fold 1). **Supplementary Table S2-2** Univariate and multivariate logistics regression analysis of the training cohort in habitat analysis (fold 3). **Supplementary Table S2-3** Univariate and multivariate logistics regression analysis of the training cohort in radiomics analysis (fold 1). **Supplementary Table S2-4** Univariate and multivariate logistics regression analysis of the training cohort in radiomics analysis (fold 2). **Supplementary Table S2-5** Univariate and multivariate logistics regression analysis of the training cohort in radiomics analysis (fold 3). **Supplementary Table S3** Diagnostic performance of the habitat clustering numbers in the training and validation cohorts. **Supplementary Table S4** Performance table of different models under three-fold cross-validation. **Supplementary Document** 1: YAML parameter file.

41747_2026_798_MOESM2_ESM

## Data Availability

The datasets used and/or analyzed during the current study are available from the corresponding author on reasonable request.

## Abbreviations

AIS: Acute ischemic stroke
ASPECTS: Alberta Stroke Program Early Computed Tomography Score
AUC: Area under the receiver operating characteristic curve
BBB: Blood–brain barrier
CEx: Contrast extravasation
CT: Computed tomography
ENI: Early neurologic improvement
EVT: Endovascular therapy
HAS: Hyperdense artery sign
HDA: Hyperdense area
HI 1: Hemorrhagic infarction type 1
HI 2: Hemorrhagic infarction type 2
HT: Hemorrhagic transformation
ICC: Intraclass correlation coefficient
NCCT: Noncontrast computed tomography
NIHSS: National Institutes of Health Stroke Scale
