# Supplementary material for: Noncontrast CT-based habitat analysis for differentiating hemorrhagic transformation from contrast extravasation after endovascular therapy of acute ischemic stroke
Source: Eur Radiol Exp. 2026 Sep 10;10:127. doi: 10.1186/s41747-026-00798-9 (PMC13562492; doi:10.1186/s41747-026-00798-9)
Supplement: Supplementary file 1 [file 41747_2026_798_MOESM1_ESM.pdf]

Noncontrast CT-based habitat analysis for differentiating hemorrhagic transformation from contrast extravasation after endovascular therapy of acute ischemic stroke

ELECTRONIC SUPPLEMENTARY MATERIAL

Eur Radiol Exp (2026) Huang Y, Tan G, Fan W et al.

SupplementaryTable S1 Scanning parameters of three CT scanners in this study.

Scanning parameters	United Imaging UCT 760	GE Healthcare Discovery™ CT750 HD	GE Healthcare Light Speed VCT 128
Slice thickness (mm)	2.5	2.5	2.5
Matrix size	512 × 512	512 × 512	512 × 512
Field of view (cm)	25.0 × 25.0	25.0 × 25.0	25.0 × 25.0
Tube current (mA)	250 - 300	240 - 250	250
Tube voltage (kVp)	120	120	120

Supplementary Table S2-1

Univariate and multivariate logistics regression analysis of the training cohort in habitat analysis (fold 1).

Characteristics	Univariate analysis		Multivariate analysis	
	**OR (95%CI)**	***P*-value**	**OR (95%CI)**	***P*-value**
Atrial fibrillation				
No	-	-		
Yes	2.24 (1.05-4.87)	0.039*	2.66 (1.12-6.57)	0.029*
Anion gap	1.12 (1.00-1.26)	0.045*	1.06 (0.94-1.21)	0.332
ENI				
No	-	0.8		
Stable	0.21 (0.06-0.71)	0.015*	0.22 (0.05-0.81)	0.027*
Yes	0.10 (0.03-0.30)	<0.001*	0.12 (0.03-0.37)	<0.001*

ENI Early neurologic improvement, *OR* Odds ratio, *CI* Confidence interval.

Supplementary Table S2-2

Univariate and multivariate logistics regression analysis of the training cohort in habitat analysis (fold 3).

Characteristics	Univariate analysis		Multivariate analysis	
	**OR (95%CI)**	***P*-value**	**OR (95%CI)**	***P*-value**
Baseline ASPECTS	0.66 (0.45-0.93)	0.023*	0.62 (0.4-0.94)	0.028*
DRT	0.99 (0.99-1.00)	0.027*	1.00 (0.99-1.00)	0.180
ENI				
No	-	-		
Stable	0.41 (0.12-1.29)	0.132	0.42 (0.09-1.74)	0.236
Yes	0.33 (0.12-0.87)	0.026*	0.30 (0.08-1.03)	0.058
FIB	0.59 (0.35-0.93)	0.035*	0.79 (0.44-1.28)	0.365
HDL	6.44 (1.27-36.98)	0.029*	3.79 (0.54-30.54)	0.188
PLR	0.99 (0.99-1.00)	0.030*	0.99 (0.99-1.00)	0.039*

ASPECTS Alberta Stroke Program Early Computed Tomography Score, *DRT* Door to

reperfusion time, *ENI* Early neurologic improvement, *FIB* Fibrinogen, *HDL* High-density lipoprotein, *PLR* Platelet-to-lymphocyte ratio, *OR* Odds ratio, *CI* Confidence interval.

Supplementary Table S2-3

Univariate and multivariate logistics regression analysis of the training cohort in radiomics analysis (fold 1).

Characteristics	Univariate analysis		Multivariate analysis	
	**OR (95%CI)**	***P*-value**	**OR (95%CI)**	***P*-value**
Baseline ASPECTS	0.66 (0.45-0.95)	0.028*	0.69 (0.46-1.00)	0.059
ENI				
No	-	-		
Stable	0.45 (0.13-1.44)	0.184	0.46 (0.13-1.49)	0.200
Yes	0.27 (0.10-0.70)	0.008*	0.31 (0.11-0.81)	0.017*

ASPECTS Alberta Stroke Program Early Computed Tomography Score, *ENI* Early neurologic improvement, *OR* Odds ratio, *CI* Confidence interval.

Supplementary Table S2-4

Univariate and multivariate logistics regression analysis of the training cohort in radiomics analysis (fold 2).

Characteristics	Univariate analysis		Multivariate analysis	
	**OR (95%CI)**	***P*-value**	**OR (95%CI)**	***P*-value**
ENI				
No	-	-	-	-
Stable	0.21 (0.06-0.71)	0.015*	0.17 (0.04-0.65)	0.012*
Yes	0.19 (0.07-0.50)	<0.001*	0.16 (0.05-0.48)	0.002*
PLR	1.00 (0.99-1.00)	0.049*	1.00 (0.99-1.00)	0.260
EVT				
3	-	-	-	-
1	2.9 (1.10-8.64)	0.040*	2.05 (0.71-6.55)	0.198
4	1.71 (0.30-8.49)	0.515	1.87 (0.31-9.96)	0.469
2	0.62 (0.08-3.13)	0.584	0.34 (0.04-2.06)	0.272
5	NA	0.992	NA	0.992

Thrombectomy status				
0	-	-	-	-
1	2.54 (1.13-6.08)	0.028*	2.03 (0.82-5.32)	0.133

ENI Early neurologic improvement, *EVT* Endovascular therapy, *PLR* Platelet-to-lymphocyte ratio; *OR* Odds ratio, *CI* Confidence interval.

Supplementary Table S2-5

Univariate and multivariate logistics regression analysis of the training cohort in radiomics analysis (fold 3).

**Characteristics**	**Univariate analysis**		**Multivariate analysis**	
	**OR (95%CI)**	***P*-value**	**OR (95%CI)**	***P*-value**
Baseline ASPECTS	0.59 (0.4-0.85)	0.006*	0.54 (0.35-0.81)	0.004*
ENI				
No	-	-		
Stable	0.11 (0.03-0.42)	0.002*	1.11 (0.38-3.00)	0.847
Yes	0.33 (0.15-0.71)	0.005*	12.84 (3.84-52.77)	<0.001*

ASPECTS Alberta Stroke Program Early Computed Tomography Score, *ENI* Early neurologic improvement, *OR* Odds ratio, *CI* Confidence interval.

Supplementary Table S3

Diagnostic performance of the habitat clustering numbers in the training and validation cohorts.

Model	Cohort	Mean AUC	95% CI	ACC	SN	SP
K=2	Training	0.82	0.74-0.89	0.75	0.63	0.80
	Validation	0.75	0.62-0.88	0.71	0.58	0.77
K=3	Training	0.84	0.77-0.91	0.75	0.73	0.75
	Validation	0.77	0.65-0.90	0.73	0.65	0.77
K=4	Training	0.90	0.84-0.95	0.75	0.90	0.67
	Validation	0.75	0.62-0.88	0.65	0.83	0.57
**K=5**	Training	**0.89**	0.83-0.95	0.80	0.78	0.81
	Validation	**0.82**	0.71-0.93	0.72	0.65	0.75
K=6	Training	0.81	0.74-0.89	0.73	0.77	0.71
	Validation	0.70	0.57-0.84	0.66	0.58	0.69

ACC Accuracy, *AUC* Area under the curve of the receiver operating characteristic curve, *Cl* Confidence interval, *SN* Sensitivity, *SP* Specificity.

Supplementary Table S4

Performance table of different models under three-fold cross-validation.

Model	Folds	Training cohort				Validation cohort			
		AUC (95%CI)	ACC	Sn	Sp	AUC (95%CI)	ACC	Sn	Sp
**Radiomics**									
Radiomics model	1	0.70 (0.60-0.80)	0.62	0.63	0.61	0.87 (0.77-0.96)	0.74	0.85	0.69
	2	0.85 (0.79-0.92)	0.75	0.83	0.72	0.73 (0.60-0.86)	0.63	0.65	0.62
	3	0.88 (0.81-0.94)	0.77	0.78	0.77	0.66(0.51-0.81)	0.59	0.55	0.60
Clinical model	1	0.63 (0.53-0.72)	0.64	0.53	0.69	0.68(0.54-0.82)	0.66	0.55	0.71
	2	0.64 (0.54-0.74)	0.73	0.38	0.89	0.66 (0.52-0.79)	0.73	0.30	0.93
	3	0.77 (0.68-0.86)	0.76	0.63	0.83	0.55 (0.38-0.72)	0.59	0.40	0.67
CT sign (HAS) model	1	0.61 (0.52-0.70)	0.58	0.68	0.54	0.60 (0.47-0.73)	0.56	0.70	0.50
	2	0.61 (0.52-0.70)	0.58	0.70	0.52	0.60 (0.47-0.73)	0.58	0.65	0.55
	3	0.60 (0.51-0.69)	0.57	0.68	0.52	0.62 (0.49-0.75)	0.59	0.70	0.53
Clinical-CT sign (HAS) model	1	0.69 (0.59-0.78)	0.55	0.83	0.42	0.76 (0.62-0.90)	0.50	0.85	0.33
	2	0.72 (0.62-0.81)	0.73	0.38	0.89	0.69 (0.55-0.84)	0.73	0.30	0.93
	3	0.80 (0.71-0.88)	0.76	0.65	0.82	0.63 (0.48-0.79)	0.63	0.50	0.70
Radiomics-Clinical model	1	0.75 (0.66-0.85)	0.50	0.98	0.27	0.94 (0.88-1.00)	0.56	1.00	0.36
	2	0.87 (0.80-0.93)	0.79	0.68	0.85	0.77 (0.64-0.89)	0.73	0.50	0.83
	3	0.91 (0.86-0.97)	0.77	0.90	0.71	0.70 (0.56-0.84)	0.52	0.75	0.42
Radiomics-CT sign (HAS) model	1	0.70 (0.60-0.81)	0.55	0.78	0.45	0.87 (0.77-0.96)	0.68	0.95	0.55
	2	0.86 (0.79-0.92)	0.75	0.63	0.81	0.73 (0.60-0.87)	0.68	0.50	0.76
	3	0.88 (0.81-0.94)	0.77	0.73	0.80	0.66 (0.51-0.81)	0.77	0.73	0.80
Combined model	1	0.76 (0.67-0.85)	0.59	0.75	0.52	0.93 (0.87-0.99)	0.59	0.75	0.52
	2	0.87 (0.81-0.93)	0.78	0.63	0.86	0.77 (0.64-0.90)	0.74	0.50	0.86
	3	0.91 (0.86-0.97)	0.82	0.83	0.82	0.70 (0.56-0.84)	0.82	0.83	0.82
**Habitat (K=5)**									
Habitat model	1	0.90 (0.84-0.95)	0.82	0.75	0.86	0.79 (0.68-0.91)	0.74	0.60	0.81
	2	0.88 (0.82-0.95)	0.78	0.78	0.78	0.82 (0.70-0.93)	0.68	0.80	0.62
	3	0.89 (0.82-0.95)	0.81	0.80	0.81	0.84 (0.74-0.94)	0.73	0.55	0.81
Clinical model	1	0.73 (0.64-0.83)	0.64	0.73	0.60	0.48 (0.32-0.63)	0.55	0.40	0.62
	2	0.77 (0.69-0.86)	0.71	0.53	0.80	0.54 (0.37-0.72)	0.63	0.40	0.74
	3	0.67 (0.57-0.77)	0.61	0.63	0.61	0.58 (0.43-0.74)	0.55	0.45	0.60

CT sign (HAS) model	1	0.63 (0.55-0.72)	0.60	0.73	0.54	0.55 (0.42-0.68)	0.53	0.60	0.50
	2	0.59 (0.50-0.68)	0.57	0.65	0.53	0.64 (0.51-0.76)	0.60	0.52	0.75
	3	0.59 (0.50-0.68)	0.56	0.68	0.51	0.63 (0.50-0.76)	0.60	0.70	0.56
Clinical-CT sign (HAS) model	1	0.77 (0.69-0.86)	0.67	0.70	0.66	0.59 (0.44-0.75)	0.60	0.45	0.67
	2	0.79 (0.71-0.87)	0.73	0.45	0.87	0.59 (0.42-0.76)	0.69	0.40	0.83
	3	0.71 (0.61-0.80)	0.67	0.58	0.71	0.66 (0.50-0.82)	0.67	0.58	0.71
Habitat-Clinical model	1	0.91 (0.86-0.96)	0.75	0.93	0.67	0.70 (0.56-0.83)	0.61	0.60	0.62
	2	0.86 (0.79-0.93)	0.79	0.65	0.86	0.77 (0.62-0.92)	0.76	0.75	0.76
	3	0.96 (0.92-0.99)	0.83	0.95	0.77	0.71 (0.57-0.85)	0.65	0.60	0.67
Habitat-CT sign (HAS) model	1	0.85 (0.78-0.93)	0.74	0.85	0.68	0.71 (0.57-0.84)	0.58	0.55	0.60
	2	0.80 (0.72-0.88)	0.72	0.58	0.79	0.79 (0.66-0.91)	0.68	0.70	0.67
	3	0.96 (0.93-0.99)	0.89	0.90	0.88	0.72 (0.58-0.85)	0.68	0.50	0.77
Combined model	1	0.91 (0.86-0.96)	0.80	0.88	0.76	0.71 (0.57-0.84)	0.63	0.55	0.67
	2	0.86 (0.79-0.93)	0.78	0.60	0.87	0.77 (0.62-0.92)	0.77	0.75	0.79
	3	0.96 (0.92-0.99)	0.89	0.90	0.88	0.71 (0.58-0.85)	0.68	0.50	0.76

ACC Accuracy, *AUC* Area under the curve of the receiver operating characteristic curve; *Cl* Confidence interval, *HAS* Hyperdense artery sign, *SN* Sensitivity, *SP* Specificity.
